# The diagnostic utility of the ultrasound TIRADS and Bethesda System of reporting thyroid cytology in thyroid nodule smaller than 4.0 cm: A retrospective analysis

**DOI:** 10.1097/MD.0000000000043264

**Published:** 2025-07-25

**Authors:** Howaida M. Hagag, Khadiga A. Ismail, Maha M. Bakhuraysah, Ali Nagi, Abdulkarim Hasan, Sabrine Ali Ahmed Ali, Tahani M. AlThagafi, Mona Abdullah M. Alsofuni, Alaa Khader S. Altalhi, Amjad Ayidh Altalhi, Salman Muidh A. Althagafi, Karim A. Ramadan, Usama M. Marzouk, Fahad A. Alghamdi, Razan Abed A. Baloush, Ahmed Abdulwahab Bawahab, Hassan A. Soltan, Mohamed Salah Elfeshawy, Tamer A.A. Samih

**Affiliations:** a Department of Clinical Laboratory Sciences, College of Applied Medical Sciences, Taif University, Taif, Saudi Arabia; b Department of General Surgery, Faculty of Medicine, Al-Azhar University, Assiut, Egypt; c Prince Mishari bin Saud Hospital, Al-Baha Health Cluster, Ministry of Health, Al-Baha, Saudi Arabia; d Department of Pathology, Faculty of Medicine, Al-Azhar University, Cairo, Egypt; e Department of Laboratory and Blood Bank, Histopathology Section, King Faisal Medical Complex, Taif, Saudi Arabia; f Department of Pathology, Alsahel Teaching Hospital, Ministry of Health, Cairo, Egypt; g Quality Department, King Faisal Medical Complex, Taif, Saudi Arabia; h Central Supply Sterilization Department, Prince Mansour Military Hospital, Taif, Saudi Arabia; i Department of Internal Medicine, Kaser Alaini Faculty of Medicine, Cairo University, Cairo, Egypt; j Department of Internal Medicine, Faculty of Medicine, Ain Shams University, Cairo, Egypt; k Department of Pathology, Faculty of Medicine, King Abdulaziz University, Jeddah, Saudi Arabia; l Department of Basic Medical Sciences, College of Medicine, University of Jeddah, Jeddah, Saudi Arabia; m Department of Radiology, Faculty of Medicine, Aswan University, Aswan, Egypt; n Department of Radiology, Faculty of Medicine, Al-Azhar University, Cairo, Egypt; o Department of Radiology, Faculty of Medicine, Benha University, Benha, Egypt.

**Keywords:** Bethesda System, FNAC, histopathology, thyroid nodules, ultrasonography

## Abstract

Thyroid nodules (TNs) are a common clinical issue, prevalent in the general population. Fine-needle aspiration cytology (FNAC) is a vital diagnostic tool for the initial evaluation of TNs. This study aimed to determine the cytopathological patterns of thyroid nodules using the Bethesda System for Reporting Thyroid Cytology (TBSRTC) and to assess the diagnostic role of both FNAC and ultrasonography in TNs <4 cm in diameter. This study included 100 FNAC samples from thyroid nodules. Patients underwent ultrasonographic examination, with findings reported according to the European Thyroid Imaging Reporting and Data System. FNAC samples were stained using Diff-Quik and Papanicolaou stains, cytologically examined, and reported according to TBSRTC. Histopathological assessment was performed using hematoxylin and eosin staining of the corresponding excised biopsies. The patterns were analyzed and the results correlated. The mean age of the participants was 46.2 ± 12.7 years, with females comprising 89% of the cases. About half (51%) of the nodules were in the right lobe. The diameter of the nodules ranged from 8 to 40 mm, with a mean diameter of 20.3 mm. Normal thyroid-stimulating hormone levels were observed in 74% of cases with no significant association between thyroid-stimulating hormone levels and nodule characteristics. Among the 100 FNACs, 54% were categorized as Bethesda II, 25% as Bethesda III, 8% as Bethesda IV, and 2% as Bethesda V. A significant correlation was found between cytopathological diagnoses and both ultrasonographic (*P* = .001) and histopathological (*P* = .05) diagnoses. We concluded that the benign conditions were the most common cytopathological findings. FNAC reporting using TBSRTC demonstrated a strong correlation with ultrasonographic and histopathological diagnoses, confirming its reliability and high diagnostic value in evaluating small thyroid nodules.

## 1. Introduction

Thyroid nodules (TNs) refer to abnormal growths of thyroid cells forming a lump within the thyroid gland.^[1]^ In recent years, TNs have garnered significant global attention due to advancements in population surveillance systems and increasing concerns regarding their financial burden. The prevalence of TNs varies widely, with the highest rates observed in low- and middle-income countries, whereas incidence is declining in high-income countries.^[2]^ Revolutionary surveillance techniques, such as fine-needle aspiration cytology (FNAC) and molecular testing, have enhanced the evaluation and management of this condition.^[2,3]^

TNs encompass a range of lesions within the thyroid gland, and their prevalence continues to rise in clinical settings. Estimates suggest a prevalence ranging from 2% to 65%, depending on the diagnostic methods employed.^[3]^

Women are more susceptible to thyroid nodules than men, indicating a potential gender-specific predisposition. The exact reasons for this disparity remain unclear; however, hormonal factors, including the influence of estrogen on thyroid function, are thought to play a role.^[4]^ Age is another significant factor, with the occurrence of thyroid nodules increasing with advancing age. This trend is attributed to prolonged exposure to environmental factors, cellular changes, and genetic susceptibilities in older individuals.^[1]^

Nodule formation may be triggered by conditions such as hypothyroidism, characterized by insufficient thyroid hormone production and elevated thyroid-stimulating hormone (TSH) levels. Alternatively, hyperthyroidism, involving excessive thyroid hormone production and suppressed TSH levels, may also contribute to nodule development. These hormonal imbalances disrupt the regulatory feedback mechanisms governing thyroid function, leading to nodule formation and enlargement.^[5]^

Thyroid nodules are classified as benign or malignant based on their pathological characteristics. Most benign thyroid nodules are small, asymptomatic, and have favorable treatment outcomes. In contrast, malignant thyroid nodules often exhibit distinctive features on ultrasound, such as irregular shape, poorly defined borders, heterogeneous composition, calcifications, hypoechogenicity, and an aspect ratio ˃1.^[6]^

From a histological perspective, benign thyroid nodules encompass a variety of lesions, including hyperplastic nodules in multinodular goiters, follicular or Hurthle cell adenomas, nodules associated with Hashimoto or Graves disease, colloid nodules, and different types of cysts (colloid, simple, or hemorrhagic).^[7]^

Thyroid cancer refers to malignant tumors arising from the thyroid parenchyma, which consists of 2 primary cell types: thyroid follicular cells and parafollicular (C-cells). Differentiated thyroid cancer, which develops from follicular cells, includes 3 major subtypes: papillary thyroid cancer, follicular thyroid cancer, and Hurthle cell cancer. Together, these account for approximately 90% to 95% of all thyroid malignancies. Medullary thyroid carcinoma, originating from C-cells, represents about 1% to 2% of thyroid malignancies, while anaplastic thyroid carcinoma comprises <1% of cases.^[1,4]^ TNs greater than or equal to 4 cm have shown a higher incidence of carcinoma and pose a more challenging preoperative cytological assessment; however, very few studies have been conducted to evaluate and differentiate between small and large-sized nodules.^[8]^

Accurate and efficient identification of nodules is critical for optimizing treatment strategies and evaluating outcomes. The majority of thyroid nodules are benign, with an estimated malignancy risk of 7% to 15%. Ultrasonography is the preferred imaging technique, offering reliable stratification of malignancy risk in thyroid nodules. This informs the need for FNAC, the most accurate preoperative diagnostic method for thyroid nodules. Complementary techniques, such as radionuclide imaging and laboratory tests, further differentiate benign from malignant lesions and aid in defining their pathologies.^[9]^

During this minimally invasive procedure, cells are aspirated from the nodule and examined microscopically. Sensitivity rates for FNAC in diagnosing thyroid malignancy range from 79% to 95%, while specificity rates vary between 72% to 100%. In comparison, histopathology demonstrates sensitivity and specificity rates of 80% to 100% and 97% to 99%, respectively. FNAC is advantageous as it avoids the need for surgical tissue sampling.^[10]^

The Bethesda System for Reporting Thyroid Cytology (TBSRTC), introduced in 2008, provides a standardized framework for reporting thyroid cytopathology. Its first edition was published in 2009, followed by updates in 2017 and 2023. TBSRTC enables cytopathologists to use a consistent reporting system, facilitating informed decision-making by healthcare providers. The system also enhances communication between pathologists and clinicians, ensuring a unified approach to FNAC result interpretation.^[11]^

The 2023 edition of TBSRTC divides thyroid cytopathology into 6 categories: I: nondiagnostic. II: benign (e.g., nodules of adenomatous or multinodular goiters, Hashimoto thyroiditis, and subacute granulomatous thyroiditis). III: follicular lesion of undetermined significance (FLUS) or atypia of undetermined significance (AUS). IV: follicular neoplasm or suspicion for follicular neoplasm (e.g., microfollicular or cellular adenomas). V: suspicious for malignancy. VI: malignant.

Each category is associated with a specific malignancy risk: 5% to 20%, 2% to 7%, 13% to 30%, 23% to 34%, 67% to 83%, and 97% to 100%, respectively.^[11]^

This study aims to determine the pathological patterns of Pap and Diff-Quik stained smears from thyroid nodule FNAC samples, as categorized by TBSRTC. It also seeks to correlate the efficacy of cytopathological diagnosis using TBSRTC with ultrasonographic and histopathological findings, thereby assessing the diagnostic accuracy of FNAC.

## 2. Materials and methods

This retrospective study included 100 patients with thyroid nodules diagnosed and managed at King Faisal Medical Complex, Taif, Saudi Arabia, during the period from January 2021 to December 2023. All participants underwent clinical and radiological examinations, in addition to FNA of their thyroid nodules. Some cases underwent surgery, including lobectomy or total/partial thyroidectomy, according to European guidelines.^[12]^ All relevant data were retrieved.

Gross examination data, including the size and location of the nodules within the thyroid glands, were collected, in addition to the demographic data, such as age and sex, that were extracted from medical records using a standardized checklist. Radiological and laboratory test results, including TSH levels and available histopathological reports, were also gathered.

FNA of the thyroid nodules was performed under ultrasound guidance. An adequate number of smears were prepared, with 1 slide air-dried and stained using Diff-Quik to assess cytoplasmic features and extracellular substances. The second slide was immediately fixed in 95% alcohol for 5 to 15 minutes, followed by Papanicolaou staining to assess nuclear features. Both slides were examined initially at low power to determine adequacy and then at high power for detailed evaluation.

Blood samples for TSH analysis were processed using the Cobas e 601 module, a fully automated machine that employs electrochemiluminescence technology for immunoassay analysis. The results were generated for interpretation, where normal values are 0.5 to 5.0 mIU/L.

Ultrasound examinations were conducted on all 100 study participants using the Philips EPIQ 7G ultrasound system by the same team or personnel with equivalent experience with remote consultation when indicated. Exclusion criteria include TN larger than 4 cm of the largest nodule (in case of multiple nodules) and cases lacking the related data or unavailable smears.

Statistical analysis was performed using Statistical Package for the Social Sciences version 23. Descriptive statistics were calculated, including the range, mean, and standard deviation (SD) for continuous variables, such as age. Categorical variables were expressed as frequencies and percentages. The Chi-square test was used to calculate the *P*-value, with a *P*-value ≤ .05 considered statistically significant. The sample size was determined using a formula for diagnostic studies, assuming a 12% prevalence of malignancy and a desired power of 85%.

A minimum of 98 patients was required to achieve a 95% confidence interval with a 7% margin of error. We included 100 patients who were diagnosed (cytologically and histologically) at our institution.

## 3. Results

**Figure 1.**
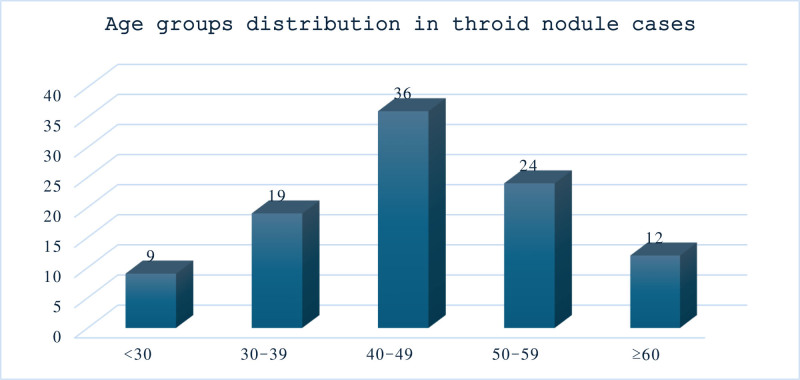
Age groups distribution (in years) in cases of thyroid nodules.

The study included a total of 100 cases with thyroid nodules. The patients’ ages ranged from 18 to 78 years, with a mean ± SD of 46.2 ± 12.7 years. The cases were divided into 5 age groups, as shown in Figure 1.

Regarding gender distribution, the majority of patients were female, accounting for 89% of the cases, with a mean age ± SD of 46.3 ± 12.8 years. Males represented only 11% of the cases, with a mean age ± SD of 45.5 ± 13.5 years. The male-to-female ratio was approximately 1:8.

TSH assessment revealed that 74% of cases had normal levels, 19% had elevated levels, and 8% had low levels. No statistically significant difference was found between TSH levels and nodule characteristics.

Gross examination findings, based on clinical and ultrasonographic assessments, showed that most nodules were located in the right lobe (51%), followed by the left lobe (38%), bilaterally (9%), and in the isthmus (2%).

The diameters of the thyroid nodules ranged from 8 to 39 mm, with a mean diameter of 20.3 mm. The nodules were categorized into 3 groups: those with a diameter < 10 mm (12%), those with a diameter between 10 to 19 mm (45%), and those with a diameter between 20 to 39 mm (43%).

The cytopathological diagnosis of FNAC of thyroid nodules, according to TBSRTC, revealed that the most common finding was benign cytology (Bethesda category II), accounting for 54% of the cases. This was followed by cytology indicating FLUS or AUS (Bethesda category III), which accounted for 25% of the cases. No cases were diagnosed with malignant cytology (Bethesda category VI), as shown in Table 1.

**Table 1:** Distribution of Bethesda categories in different age groups.

Age groups	Cytopathological diagnosis according to TBSRTC						
	Bethesda category I n = 11 (%)	Bethesda category II n = 54 (%)	Bethesda category III n = 25 (%)	Bethesda category IV n = 8 (%)	Bethesda category V n = 2 (%)	x^2^	*P* value
<30 years	0 (0%)	5 (9%)	1 (4%)	2 (25%)	1 (50%)	13.86	.086
30–39 years	3 (27%)	8 (15%)	7 (28%)	1 (12.5%)	0 (0%)		
40–49 years	5 (45)	24 (44)	6 (24%)	1 (12.5%)	0 (0%)		
50–59 years	2 (18)	11 (20)	7 (28%)	3 (37.5%)	1 (50%)		
≥60 years	1 (9)	6 (11%)	4 (16%)	1 (12.5%)	0 (0%)		

Non statistically significant.

Regarding the distribution of Bethesda categories according to patient gender, all Bethesda categories were more prevalent in females than males. However, in category V, the distribution was equal between genders, as shown in Table 2.

**Table 2:** Distribution of the Bethesda categories among patient’s gender.

Gender	Cytopathological diagnosis according to TBSRTC						
	Bethesda category I n = 11 (%)	Bethesda category II n = 54 (%)	Bethesda category III n = 25 (%)	Bethesda category IV n = 8 (%)	Bethesda category V n = 2 (%)	x^2^	*P* value
Females	10 (91%)	47 (87%)	24 (96%)	7 (87.5%)	1 (50%)	4.6	.327
Males	1 (9)	7 (13%)	1 (4%)	1 (12.5%)	1 (50%)		

Non statistically significant.

The relationship between TSH level and cytopathological diagnosis (Bethesda categories) is shown in Table 3.

**Table 3:** The relation between cytopathological diagnosis (Bethesda categories) and TSH level.

TSH level	Cytopathological diagnosis according to TBSRTC						
	Bethesda category I n = 11 (%)	Bethesda category II n = 54 (%)	Bethesda category III n = 25 (%)	Bethesda category IV n = 8 (%)	Bethesda category V n = 2 (%)	x^2^	*P* value
Normal	10 (91%)	40 (74%)	17 (68%)	5 (62.5%)	2 (100%)	3.61	.982
High	1 (9%)	10 (18.5)	6 (24%)	2 (25%)	0 (0%)		
Low	1 (9%)	4 (7.5%)	2 (8%)	1 (12.5%)	0 (0%)		

Non statistically significant.

The relationship between the diameter of thyroid nodules and the cytopathological diagnosis (Bethesda categories) is shown in Table 4.

**Table 4:** The relation between cytopathological diagnosis (Bethesda categories) and thyroid nodules diameter.

Subgroup of nodule diameter	Cytopathological diagnosis according to TBSRTC						
	Bethesda category I n = 11 (%)	Bethesda category II n = 54 (%)	Bethesda category III n = 25 (%)	Bethesda category IV n = 8 (%)	Bethesda category V n = 2 (%)	x^2^	*P* value
<10 mm	2 (18%)	4 (7.4%)	5 (20%)	1 (12.5%)	0 (0%)	13.37	.037*
10–19 mm	5 (45%)	19 (35.2%)	13 (52%)	6 (75%)	2 (100%)		
≥20 mm	4 (36%)	31 (57.4%)	7 (28%)	1 (12.5%)	0 (0%)		

* Statistically significant if cases of category V are not included.

Regarding the correlation between ultrasonographic and cytopathological diagnoses, in Bethesda category II cases: 3.7% were classified as TIRADS I, 50% as TIRADS II, 29.6% as TIRADS III, and 16.7% as TIRADS IV by ultrasound.

**Figure 2.**
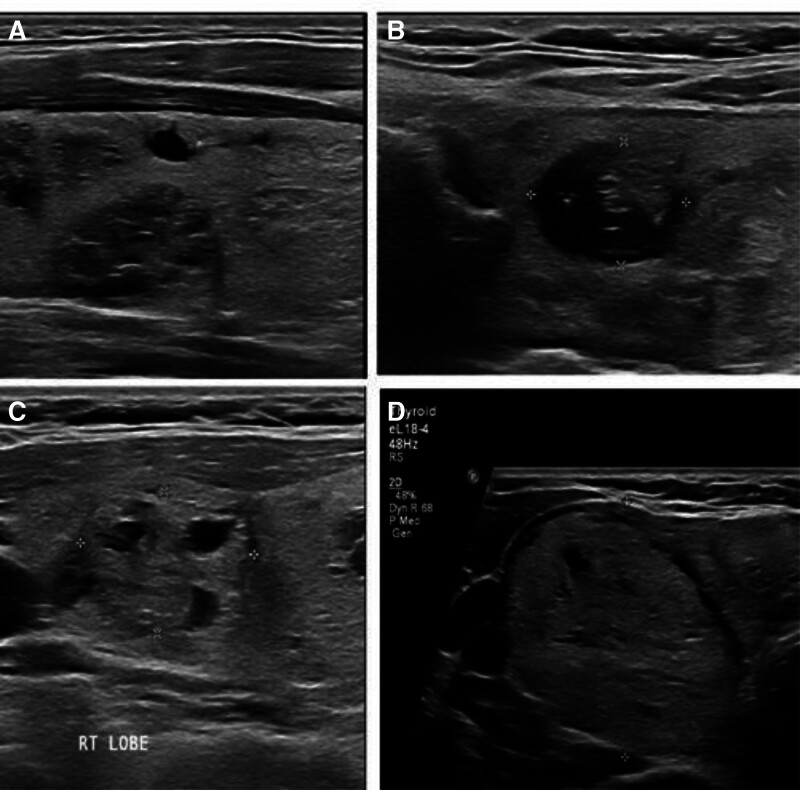
US pictures: (A) transverse US image shows solid hyperechoic nodule with ill-defined border and small lobulation EU-TIRADS 4, small cystic nodule seen anteriorly. (B) Transverse US image shows solid hyperechoic nodule with well-defined border and halo sign EU-TIRADS. (C) Transverse US image shows mixed solid and cystic nodules with well-defined border and small lobulation. (D) Transverse US image shows predominant solid nodules with small component, well defined border and taller than wider EU-TIRADS 4, smaller solid hyperechoic nodule seen at the isthmus EU-TIRADS 3.

The cases of Bethesda category III: 4% were diagnosed as TIRADS I, 16% were diagnosed as TIRADS II, 52% were diagnosed as TIRADS III, and 28% were diagnosed as TIRADS IV. In Bethesda category IV, 12.5% of cases were diagnosed as TIRADS II and 12.5% were diagnosed as TIRADS V, 37.5% were diagnosed as TIRADS III, and 37.5% were diagnosed as TIRADS IV by ultrasound. A 50% of cases in Bethesda V category were diagnosed as TIRADS IV and 50% of cases were diagnosed as TIRADS V by ultrasound as shown in Table 5 and Figure 2.

**Table 5:** Comparison of cytopathological diagnosis (Bethesda categories) and ultrasonographic diagnosis of thyroid nodules.

Ultrasonographic diagnosis	Cytopathological diagnosis according to TBSRTC						
	Bethesda category I n = 11 (%)	Bethesda category II n = 54 (%)	Bethesda category III n = 25 (%)	Bethesda category IV n = 8 (%)	Bethesda category V n = 2 (%)	x^2^	*P* value
TIRADS I	0 (0%)	2 (3.7%)	1 (4%)	0 (0%)	0 (0%)	39.50	.001*
TIRADS II	7 (63.6%)	27 (50%)	4 (16%)	1 (12.5%)	0 (0%)		
TIRADS III	3 (27.4%)	16 (29.6)	13 (52%)	3 (37.5)	0 (0%)		
TIRADS IV	0 (0%)	9 (16.7%)	7 (28%)	3 (37.5)	1 (50%)		
TIRADS V	1 (9%)	0 (0%)	0 (0%)	1 (12.5%)	1 (50%)		

EU-TIRADS 1: normal; TIRADS 2: benign; TIRADS 3: low risk; TIRADS 4: intermediate risk; TIRADS 5: high risk.

* Statistically significant.

The histopathological diagnosis was done for 51% of cases of thyroid nodules. Benign diagnosis included nodular thyroid hyperplasia (15 cases, 29.4%), nodular goiter (8 cases, 15.7%), thyroiditis (7 cases, 13.7%), and Hurthle cell adenoma (3 cases, 5.9%). Malignant diagnosis included papillary carcinoma (6 cases, 11.8%) and noninvasive follicular neoplasm with papillary-like nuclear features (9 cases, 17.6 %), as shown in Figure 4.

Regarding the comparison between cytopathological diagnosis (Bethesda categories) and histopathological diagnosis for the 51 cases that underwent surgical excision (18 cases were previously diagnosed as Bethesda category II, 18 cases as category III, and 15 cases as category IV).

Category II: of the 18 cases, 16 (88.9%) were diagnosed histopathologically as benign, while 2 (11.1%) were diagnosed as malignant.

Category III: of the 18 cases, 13 (72.2%) were diagnosed histopathologically as benign, and 5 (27.8%) were diagnosed as malignant.

Category IV: of the 15 cases, 9 (60%) were diagnosed histopathologically as benign, and 6 (40%) were diagnosed as malignant, as shown in Tables 6 and 7.

**Table 6:** Comparison of cytopathological and Histopathological diagnosis for thyroid nodule.

Histopathological diagnosis	Cytopathological diagnosis according to TBSRTC				
	Bethesda II n = 18 (%)	Bethesda III n = 18 (%)	Bethesda IV n = 15 (%)	x^2^	*P* value
Benign	16 (88.9%)	13 (72.2%)	9 (60%)	3.71	.05*
Malignant	2 (11.1%)	5 (27.8%)	6 (40%)		

* Statistically significant.

**Table 7:** Histopathological diagnosed malignancies among different categories of the Bethesda System for reporting thyroid cytopathology in cases of thyroid nodules under study.

Cytopathological diagnosis (Bethesda categories)	Percentage of malignancy by histopathology
Benign (Category II)	11.1%
FLUS/AUS (Category III)	27.8%
FN/SFN (Category IV)	40%

AUS = atypia of undetermined significance, FLUS = follicular lesion of undetermined significance, FN = follicular neoplasm.

**Figure 3.**
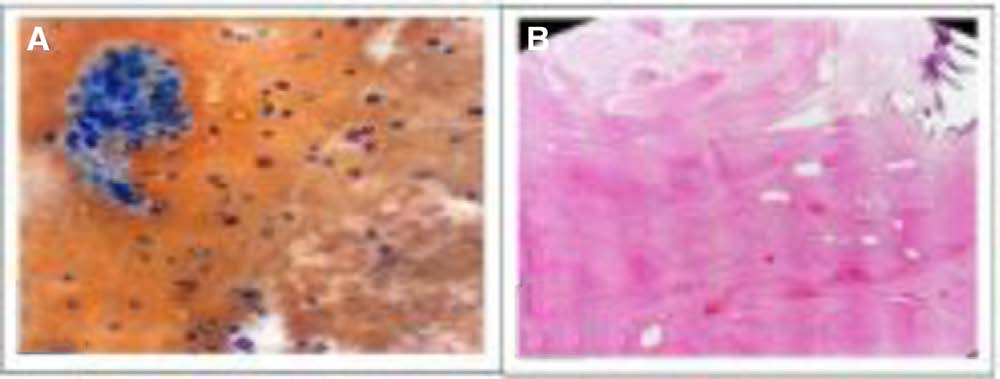
(A) Pap-stained cytology smear showing follicular cellular pattern with scanty colloid suggestive FLUS (40× magnification). (B) Corresponding histopathogical H&E-stained section showing variable size follicles filled with colloid with areas of cystic changes and calcification diagnosed as nodular thyroid hyperplasia with marked secondary changes (4× magnification). H&E = hematoxylin and eosin.

**Figure 4.**
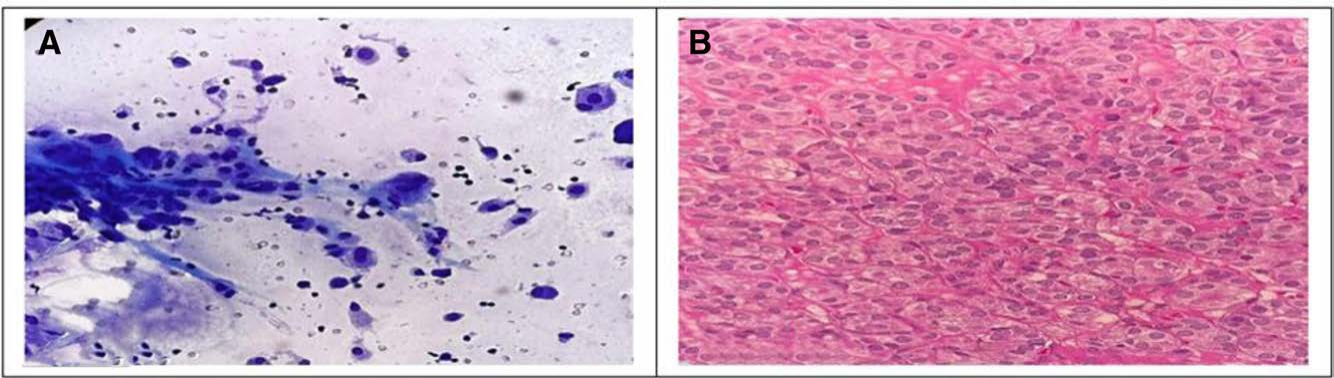
(A) Diff quick stained cytology smear showing cluster of cells with few atypical features, the background of chronic inflammatory infiltrates suggestive AUS (40× magnification). (B) Corresponding histopathological H&E-stained section showing follicles lined by cells with atypical nuclei, nuclear grooves, and intranuclear inclusion, diagnosed as noninvasive thyroid tumor with papillary like nuclear features (40× magnification).

**Figure 5.**
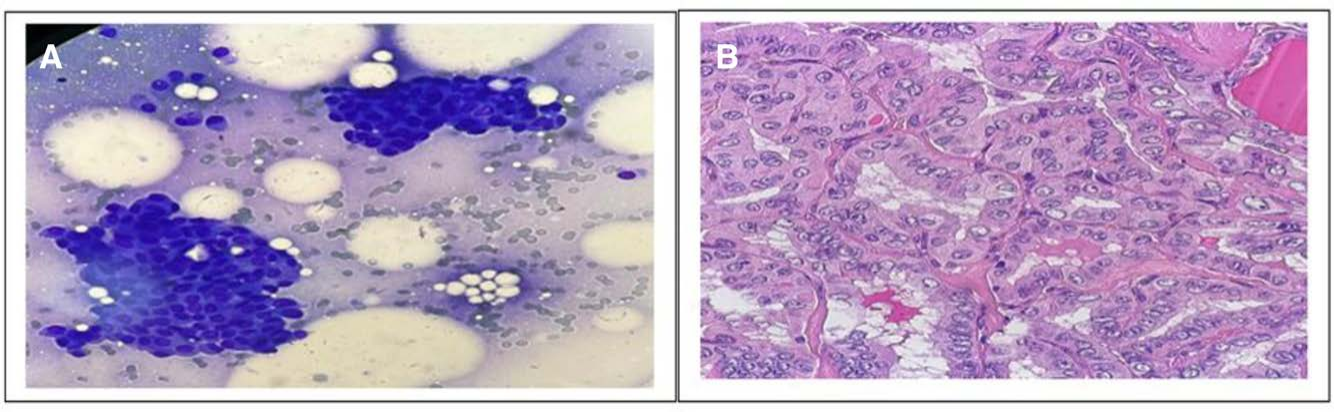
(A) Diff quick stained cytology smear showing cluster of atypical cells with follicular pattern suggestive follicular neoplasm (40× magnification). (B) Corresponding histopathogical H&E-stained section showing follicular and papillary architecture lined by cells with papillary nuclear features (pale chromatin, micronucleoli, and nuclear groove), diagnosed papillary carcinoma (follicular variant) (40× magnification).

Figures 3 to 5 show photomicrographs from microscopic examination of Pap and Diff quick stained FNAC smears of thyroid nodules and hematoxylin and eosin-stained paraffin sections from corresponding excised specimens.

## 4. Discussion

Thyroid nodules are commonly encountered in the general population, particularly in females, where the reported female-to-male ratio is about 3 to 4:1, that may be due the high estrogen levels raising the risk for TNs.^[13,14]^ Our study aligns with this result, with 89 out of 100 cases diagnosed as thyroid nodules being female, and only 11% of cases being male. The male-to-female ratio was approximately 1:8, which is consistent with a recent study conducted in Najran by Alqahtani^[15]^ involving 1353 thyroid nodule cases, where 84.1% were females and 15.9% were males. Similarly, a study in Beijing by Xu et al^[4]^ involving 19,699 patients showed 13,030 females and 6669 males.

Several studies suggest a significant correlation between age and thyroid nodules. Thyroid nodules tend to increase with age due to the accumulation of reactive oxygen species, leading to cellular changes and alterations in the thyroid parenchyma.^[1]^ In our study, patients’ ages ranged from 18 to 78 years, with a mean age ± SD of 46.2 ± 12.7. The mean age in female patients was 46.3 ± 12.8, and in male patients, it was 45.5 ± 13.53. The age group most affected by thyroid nodules was 40 to 49 years, accounting for 36% of cases, followed by 24% in the 50 to 59 years age group. This finding is consistent with a study by Saeed et al^[16]^ from Saudi Arabia but higher than the average age reported by Nagaty et al^[17]^ in Egypt.

In terms of the location of thyroid nodules, our study found 51% in the right lobe, 39% in the left lobe, 9% bilateral, and 2% in the isthmus. This finding is consistent with another study by Xu et al,^[4]^ which reported similar results. The higher frequency of nodules in the right lobe may be attributed to the natural size difference between the thyroid lobes, as the right lobe is usually 20% larger than the left.^[1]^ However, Zhang et al^[18]^ found a higher frequency of nodules in the left lobe, with fewer in the isthmus.

TBSRTC standardizes FNA reporting and has high predictive value and reproducibility. In our study, Bethesda category II (benign) was the most common diagnosis (54%), followed by Bethesda category III (AUS/FLUS) at 25%. Bethesda category I (ND) accounted for 11%, while categories IV (follicular neoplasm) and V (suspicious for malignancy) were observed in 8% and 2% of cases, respectively. This distribution aligns with findings from studies by Al-Hamadani and Hussein^[19]^ and Anand et al,^[20]^ where benign thyroid nodules were the most common.

TSH measurement is essential in the initial workup of thyroid nodules. In our study, 74% of cases had normal TSH levels, 19% had high levels, and 7% had low levels. These results are consistent with a study by Li et al.^[21]^ However different results are found by several researchers who reported a significant difference in TSH level and the nature of the resected nodule^[22,23]^

In attempting to assess the relationship between Bethesda categories and risk factors such as age and gender, we found no statistically significant differences in the distribution of Bethesda categories across age groups or gender. This finding may be attributed to the limited sample size and the short duration of the study. A previous study by Achkar et al^[24]^ also found no correlation between Bethesda classification and gender, and a negative correlation with age.

Although TSH levels are important for diagnosing thyroid nodules, our study did not find a statistically significant relationship between TSH levels and Bethesda categories. This result concurs with a study by Poudel et al,^[25]^ which also found no significant association between TSH levels and Bethesda categories. However, it is important to note that TSH levels are frequently used in combination with FNAC to help correlate cytology with thyroid function.

Regarding the relationship between nodule size and Bethesda category, we found a statistically significant (*P* value = .037) association between most Bethesda categories (II, III, and IV) and nodules measuring 10 to 19 mm or ≥ 20 mm in diameter.

We also evaluated the correlation between cytopathological diagnoses (Bethesda categories) and both ultrasonographic and histopathological diagnoses to assess the diagnostic accuracy of FNAC. When comparing FNAC and ultrasound diagnoses in our study, we found a significant correlation (*P* = .001), which suggests that ultrasound can aid in improving the accuracy of FNAC results. Ultrasound diagnoses TIRADS V, which indicate a malignant risk of over 80%, were found in 50% of cases diagnosed as Bethesda category V, which also carries a malignant risk of 67% to 83%. Conversely, ultrasound diagnoses (TIRADS II), indicating a 0% risk of malignancy, were found in 63% of cases diagnosed as Bethesda category II, which has a very low risk of malignancy. These findings are in agreement with a retrospective study conducted by Al-Ghanimi et al,^[26]^ which demonstrated that ultrasonography is a reliable tool for identifying thyroid nodules and can improve decision-making regarding biopsy.

Histopathological evaluation of the 51 cases that underwent excision revealed that the most common benign diagnoses were nodular thyroid hyperplasia (29.4%), nodular goiter (15.7%), and thyroiditis (13.7%). The malignant diagnoses included noninvasive follicular neoplasm with papillary-like nuclear features (23.5%) and papillary carcinoma (11.8%).

When comparing FNAC cytology with histopathological results, we found that 88.9% of benign cases (Bethesda II) were confirmed as benign by histopathology, while 11.1% were diagnosed as malignant. For Bethesda category III, 72.2% were benign, and 27.8% were malignant, while in Bethesda category IV, 60% were benign, and 40% were malignant. The correlation between cytopathological and histopathological diagnoses was statistically significant (*P* = .05). Our findings are in agreement with a study by Bayrak and Eruyar,^[27]^ which showed similar results in their comparison of FNAC and histopathology in Bethesda categories III and IV.

In our study, the malignancy rate in histopathological examination of benign FNAC diagnoses (Bethesda II) was 11.1%, which is higher than the reported risk of 2% to 7% for Bethesda category II cytology.^[28,29]^ This discrepancy can be attributed to false-positive diagnoses, as described by Alzahrani and Alghamdi in a study from Saudi Arabia.^[30]^ False positives were often due to secondary changes or cystic degeneration observed in histopathology.

Regarding nodule size, which may be used to predict malignancy risk, the most frequent nodule diameter detected by ultrasound in our study was 10 to 19 mm (45%), followed by ≥ 20 mm (43%). These findings differ from a study by Tran et al,^[1]^ which found that three-fourths of their cases had nodules with diameters ≤ 10 mm, but they assessed all thyroid nodules not only the patients who experienced cytological aspiration. Magister et al^[31]^ studied the patients who underwent FNA from TNs and found that the nodules ranged from 0.5 to 8.8 cm with a mean size of 2.0 cm and about 80 % of cases recorded TN larger than 3 cm.

Many of such previous studies concluded that the larger nodular size, the more probability of malignancy and suggesting reduced diagnostic accuracy of FNA in nodules > 4 cm in diameter.^[31–33]^

In this study, FNAC demonstrates a significant diagnostic utility in assessing thyroid nodules <4 cm in diameter, although there are some false positives and negatives. These discrepancies may be due to sampling errors or suboptimal slide preparation. A combination of experienced ultrasound operators and cytopathologists can help reduce false negatives, and careful attention to sampling technique can minimize false positives.

Limitations of this study include lack of long-term follow-up data and unavailable cases of FNA Bethesda VI, possibly due to submitting to surgery before performing FNA of the clinically and radiologically highly suspicious cases or preference of the patients to be managed in a specialized cancer institution.

## 5. Conclusion

Thyroid nodules are common, especially among females and in the fourth decade of life. Benign conditions are the most common cytopathological diagnosis for thyroid nodules using TBSRTC. FNA is a reliable and effective procedure for evaluating small thyroid nodules, with high diagnostic accuracy. FNAC reporting using TBSRTC is highly correlated with ultrasonographic diagnosis, enhancing the clinical management of thyroid nodules.

## Acknowledgments

Authors would like to thank Taif University Project No. (TU-DSPP-2024-81), KFMC histopathology lab staff, and sonography technicians for providing the facilities and the support during the performance of this research.

## Author contributions

**Conceptualization:** Howaida M. Hagag, Khadiga A. Ismail, Maha M. Bakhuraysah, Ali Nagi, Abdulkarim Hasan, Sabrine Ali Ahmed Ali, Tahani M. AlThagafi, Mona Abdullah M. Alsofuni, Alaa Khader S. Altalhi, Salman Muidh A. AlThagafi, Karim A. Ramadan, Fahad A. Alghamdi, Razan Abed A. Baloush, Ahmed Abdulwahab Bawahab, Hassan A. Soltan, Tamer A.A. Samih .

**Data curation:** Howaida M. Hagag, Khadiga A. Ismail, Maha M. Bakhuraysah, Sabrine Ali Ahmed Ali, Tahani M. AlThagafi, Mona Abdullah M. Alsofuni, Alaa Khader S. Altalhi, Salman Muidh A. AlThagafi, Amjad Ayidh Altalhi, Ahmed Abdulwahab Bawahab.

**Formal analysis:** Howaida M. Hagag, Ali Nagi, Abdulkarim Hasan, Sabrine Ali Ahmed Ali, Tahani M. AlThagafi, Alaa Khader S. Altalhi, Razan Abed A. Baloush, Hassan A. Soltan, Mohamed Salah Elfeshawy, Tamer A.A. Samih .

**Funding acquisition:** Khadiga A. Ismail.

**Investigation:** Howaida M. Hagag, Ali Nagi, Abdulkarim Hasan, Salman Muidh A. AlThagafi, Amjad Ayidh Altalhi, Karim A. Ramadan, Razan Abed A. Baloush, Hassan A. Soltan .

**Methodology:** Howaida M. Hagag, Maha M. Bakhuraysah, Sabrine Ali Ahmed Ali, Tahani M. AlThagafi, Amjad Ayidh Altalhi, Karim A. Ramadan, Usama M. Marzouk, Fahad A. Alghamdi, Razan Abed A. Baloush, Mohamed Salah Elfeshawy, Tamer A.A. Samih.

**Resources:** Abdulkarim Hasan, Ahmed Abdulwahab Bawahab, Mohamed Salah Elfeshawy.

**Software:** Usama M. Marzouk, Fahad A. Alghamdi, Ahmed Abdulwahab Bawahab.

**Supervision:** Howaida M. Hagag.

**Validation:** Abdulkarim Hasan.

**Visualization:** Howaida M. Hagag, Khadiga A. Ismail, Mona Abdullah M. Alsofuni, Usama M. Marzouk, Hassan A. Soltan, Mohamed Salah Elfeshawy, Tamer A.A. Samih.

**Writing – original draft:** Howaida M. Hagag, Khadiga A. Ismail, Maha M. Bakhuraysah, Abdulkarim Hasan, Sabrine Ali Ahmed Ali, Tahani M. AlThagafi, Mona Abdullah M. Alsofuni, Amjad Ayidh Altalhi, Karim A. Ramadan, Usama M. Marzouk, Razan Abed A. Baloush, Ahmed Abdulwahab Bawahab, Hassan A. Soltan.

**Writing – review & editing:** Howaida M. Hagag, Khadiga A. Ismail, Maha M. Bakhuraysah, Ali Nagi, Abdulkarim Hasan, Sabrine Ali Ahmed Ali, Mona Abdullah M. Alsofuni, Alaa Khader S. Altalhi, Salman Muidh A. AlThagafi, Amjad Ayidh Altalhi, Karim A. Ramadan, Usama M. Marzouk, Fahad A. Alghamdi, Razan Abed A. Baloush, Ahmed Abdulwahab Bawahab, Hassan A. Soltan, Mohamed Salah Elfeshawy, Tamer A.A. Samih .
